# *Rickettsia asembonensis* Characterization by Multilocus Sequence Typing of Complete Genes, Peru

**DOI:** 10.3201/eid2405.170323

**Published:** 2018-05

**Authors:** Steev Loyola, Carmen Flores-Mendoza, Armando Torre, Claudine Kocher, Melanie Melendrez, Alison Luce-Fedrow, Alice N. Maina, Allen L. Richards, Mariana Leguia

**Affiliations:** US Naval Medical Research Unit No. 6, Lima, Peru (S. Loyola, C. Flores-Mendoza, A. Torre, C. Kocher, M. Leguia);; Pontificia Universidad Católica del Perú, Lima (A. Torre, M. Leguia);; Walter Reed Army Institute of Research, Silver Spring, Maryland, USA (M. Melendrez);; Naval Medical Research Center, Silver Spring (A. Luce-Fedrow, A.N. Maina, A.L. Richards)

**Keywords:** Rickettsia asembonensis, multilocussequence typing, Peru, next-generation sequencing, Rickettsia, vector-borne infections, fleas, zoonoses, ectoparasites, parasites

## Abstract

While studying rickettsial infections in Peru, we detected *Rickettsia asembonensis* in fleas from domestic animals. We characterized 5 complete genomic regions (17kDa, *gltA*, *ompA*, *ompB*, and *sca4*) and conducted multilocus sequence typing and phylogenetic analyses. The molecular isolate from Peru is distinct from the original *R. asembonensis* strain from Kenya.

*Rickettsia asembonensis* belongs to a group of *R. felis*–like organisms (RFLOs) that are similar, yet distinct, from their closest known relative, *R. felis* (*1*,*2*). Although *R. felis* causes disease in humans (*3*), the pathogenicity of RFLOs remains unknown (*1*,*4*,*5*). *R. asembonensis* was initially identified in domestic fleas from Kenya (*1*). Subsequently, reports from the Americas, Asia, and Africa established that *R. asembonensis* is ubiquitous and closely associated with human habitats because of its arthropod hosts (*4*–*7*). However, reports of *R. asembonensis* rarely include robust genomic information needed to establish degrees of genetic diversity. Consequently, many rickettsial infections remain underdiagnosed, even when prevalence is high (*8*). We recently described *R. asembonensis* in multiple ectoparasites (*Ctenocephalidesfelis* fleas and *Rhipicephalussanguineus* ticks) collected in the Peruvian Amazon (*9*). Here, we detail multilocus sequence typing of a single molecular isolate using next-generation sequencing data for 5 complete genomic regions, including conserved (17kDa and *gltA*) and variable (*ompA*, *ompB*, and *sca4*) genes.

The internal review board of the US Naval Medical Research Unit No. 6 and the Institutional Animal Care and Use Committee approved the study protocol in compliance with all applicable regulations. Genomic DNA was mechanically extracted from half of a single *C. felis* flea as described (*9*) and fragmented by Bioruptor (Diagenode, Denville, NJ, USA). Fragmented DNA served as template to prepare IonPGM libraries using IonPlus Fragment Library Kits (ThermoFisher, Lima, Peru) according to the manufacturer’s directions. We conducted quality control using Bioanalyzer High Sensitivity chips (Agilent, Lima, Peru). We prepared libraries for sequencing using IonPGM Template OT2 200 Kits (ThermoFisher, Lima, Peru) and conducted sequencing on 318 chips using IonPGM Sequencing 200 Kits v2 (ThermoFisher). We processed raw data by reference mapping against NMRCii from Kenya (*10*). Of the 20,575,878 shotgun sequencing reads generated, ≈12% matched Rickettsiaceae.

Comparison of the consensus sequences we generated (GenBank accession nos. KY650696–KY650700) with those of strain NMRCii (GenBank accession no. JWSW01000078.1) (*10*) indicates high identity at the nucleotide (99.8%–100.0%) and amino acid (99.6–100.0%) levels (Table). As expected, conserved genes (17kDa and *gltA*) showed fewer substitutions than variable genes (*ompA*, *ompB*, and *sca4*). The 17kDa gene exhibited no mutations along its 480-nt open reading frame (ORF), whereas the *gltA* gene exhibited 3 mutations along its 1,314-nt ORF. Two mutations in *gltA* encoded silent changes, whereas the third encoded a conservative lysine-to-glutamic acid change at position 290. In the variable group, *ompB* exhibited no mutations along its 4,947-nt ORF; *omp*A and *sca4* exhibited 5 each. *ompA* had 2 conservative changes (leucine-to-valine at position 1454 and valine-to-alanine at position 1627) and 2 nonconservative changes (tyrosine-to-aspartic acid at position 162 and arginine-to-glycine at position 1280). *sca4* had 1 conservative change (glutamine-to-histidine at position 608) and 3 nonconservative changes (leucine-to-proline at position 128, arginine-to-glycine at position 754, and isoleucine-to-threonine at position 831). On the basis of these data, we conclude that the Peru molecular isolate is distinct from the original Kenya strain.

**Table:** Multilocus sequence typing analysis of complete genes from Peru *Rickettsia asembonensis* molecular isolate VGD7*

Changes	Conserved genes (GenBank accession no.)			Variable genes (GenBank accession no.)		
	17-kDa (KY650696)	*gltA* (KY650697*)*		*ompA* (KY650698)	*ompB* (KY650699)	*sca4* (KY650700)
Genome						
Complete ORF, nt	480	1,314		5,076	4,947	3,033
Identity, %	100	99.8		99.9	100	99.8
Mutations	None	C 138 T		T 484 G	None	T 383 C
		T 537 C		C 828 A		T 807 C
		A 868 G		A 3838 G		G 1824 T
				C 4360 G		A 2260 G
				T 4880 C		T 2492 C
Total changes	0	3		5	0	5
Protein						
Complete protein, aa	160	438		1,692	1,649	1,011
Identity, %	100	99.8		99.8	100	99.6
Mutations	None	K 290 E		Y 162 D	None	L 128 P
				R 1280 G		Q 608 H
				L 1454 V		R 754 G
				V 1627 A		I 831 T
Total changes	0	1		4	0	4

*Comparisons were made against reference strain NMRCii from Kenya (GenBank accession no. JWSW01000078.1). Blank cells indicate no additional mutations or changes to report. ORF, open reading frame.

To further characterize the Peru isolate, we conducted phylogenetic analysis using the conserved *gltA* gene. Although reference sequences are available for multiple Rickettsiaceae, *R. asembonensis* sequences are limited in number and length (Technical Appendix Table). Nevertheless, we constructed a phylogenetic tree using almost the complete *gltA* gene (1,068 [81%] nt of the ORF). As expected, the Peru isolate groups with RFLOs, including other *R. asembonensis* isolates and *R. senegalensis* (Technical Appendix Figure ,panel A). Construction of an additional tree using only 348 nt of *gltA* sequence available for an increased number of isolates (Technical Appendix Table) enabled us to confirm placement and relationship with other strains from the Americas (Technical Appendix Figure, panel B). This tree focuses exclusively on the transitional group and includes partial *R. asembonensis* references from Brazil, Colombia, and Costa Rica that were not available for inclusion in the 1,068-nt *gltA* tree. The Peru isolate clearly groups with other American isolates, and this subgroup is distinct from the original Kenya strain.

*R. asembonensis* is a new species (*2*) with potential as a ubiquitous human pathogen. Despite worldwide distribution, whether *R. asembonensis* and other RFLOs are pathogenic to humans, as is their closest relative *R. felis*, remains unknown. Complete genomic data, which are largely lacking from public repositories, are required to assess genetic diversity. Using next-generation sequencing, we generated complete sequences for 2 conserved (17 kDa and *gltA*) and 3 variable (*ompA*, *ompB*, and *sca4*) genes of an *R. asembonensis* molecular isolate from Peru. Although characterization of 1 isolate is not sufficient to evaluate strain diversity within Peru, much less among American strains, these sequences represent a major contribution toward the expansion of availability of much needed genomic information. Our multilocus sequence typing and phylogenetic analyses indicate that the Peru isolate is closer to American strains than to the original strain from Kenya. Characterization of additional isolates, derived from a variety of ectoparasites in which *R. asembonensis* has been detected, is needed to further validate our findings and to conduct in-depth diversity studies. In turn, these results should help decrease the chronic underdiagnosis of rickettsial diseases throughout the Americas.

Technical Appendix Reference sequences used for phylogenetic analysis of Peru *Rickettsia asembonensis* molecular isolate.
