# Supplementary material for: Rickettsia asembonensis Characterization by Multilocus Sequence Typing of Complete Genes, Peru
Source: Emerg Infect Dis. 2018 May;24(5):931–3. doi: 10.3201/eid2405.170323 (PMC5938772; doi:10.3201/eid2405.170323)
Supplement: Technical Appendix — Reference sequences used for phylogenetic analysis of Peru Rickettsia asembonensis molecular isolate. [file 17-0323-Techapp-s1.pdf]

Article DOI: https://doi.org/10.3201/eid2405.170323

Rickettsia asembonensis Characterization by Multilocus Sequence Typing of Complete Genes, Peru

Technical Appendix

Technical Appendix Table. Reference sequences used for phylogenetic analysis of *Rickettsia asembonensis*, Peru

	gltA conserved gene	
Rickettsia species (strain name)	GenBank accession no.	Length, nt
R. rickettsii	KF742602.1	1,068
R. peacockii	DQ100162.1	1,233
R. honei	U59726.1	1,234
R. conorii	U59728.1	1,234
R. africae	U59733.1	1,234
R. parkeri	U59732.1	1,234
R. mongolotimonae	DQ097081.1	1,234
R. slovaca	U59725.1	1,234
R. heliongjangensis	AF178034.2	1,238
R. japonica	U59724.1	1,234
R. raoultii	DQ365804.1	1,250
R. aeschlimannii	AY259084.1	1,159
R. massiliae	U59719.1	1,234
R. rhipicephali	U59721.1	1,234
R. montanensis	U74756.1	1,234
R. felis (URRWXCal2)	NC_007109.1	1,308
R. asembonensis (NMRCii)	JWSW01000078.1	1,410
R. asembonensis (Tapes)	KX196267.1	1,137
R. asembonensis (Derrubadas-RS)	KX533943.1	348
R. asembonensis (9AL)	KJ569090.1	350
R. asembonensis (0-TP-1)	KX544807.1	349
R. asembonensis (1-TB-1)	KX544808.1	349
R. asembonensis (6-CP-4–3)	KX544810.1	349
R. asembonensis (6-CP-4–4)	KX544811.1	349
Candidatus R. senegalensis	KF666472.1	1,250
R. akari	U59717.1	1,234
R. australis	U59718.1	1,234
R. typhi	U59714.1	1,234
R. prowazekii (Madrid E)	CP004888.1	1,311

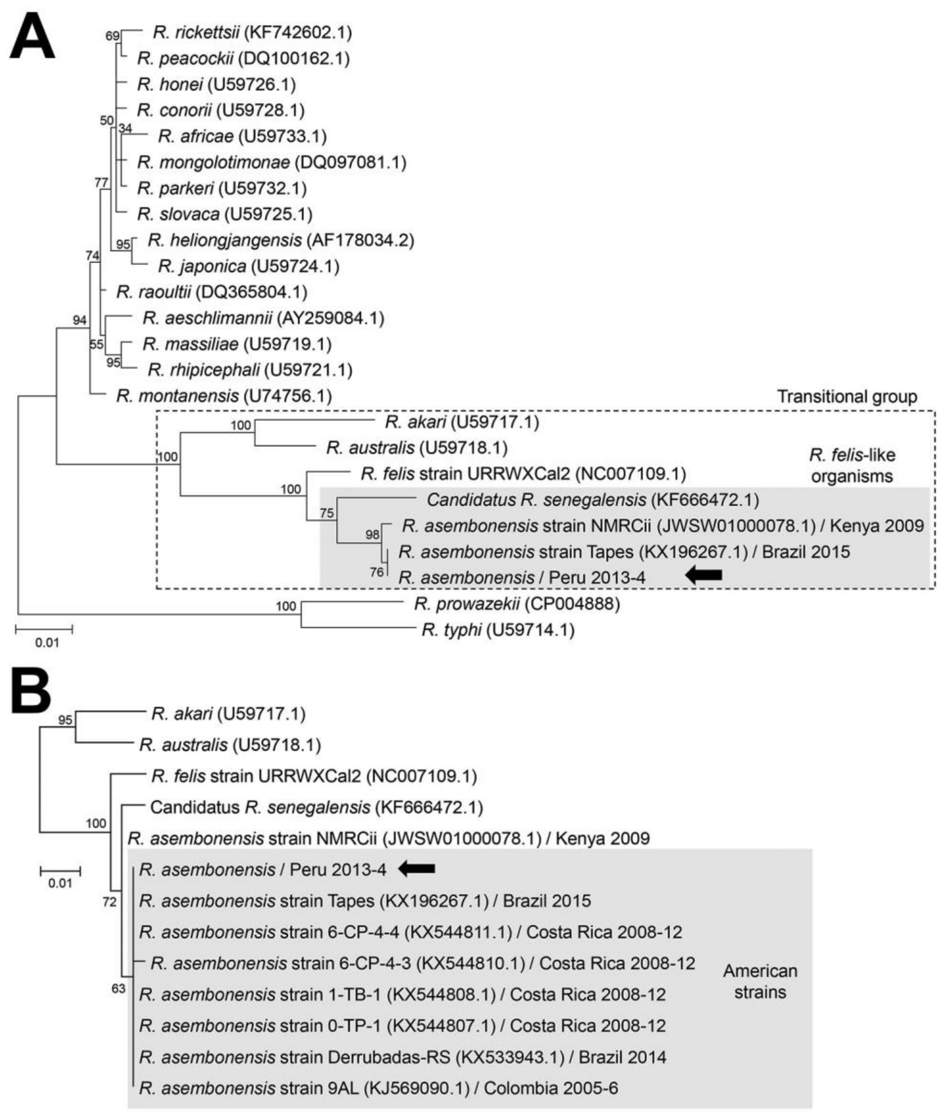

Technical Appendix Figure. Phylogenetic analysis of *gltA* using A) 1,068 (81%) nt and B) 348 (27%) nt of the open reading frame. Alignments were generated in MUSCLE (http://www.drive5.com/muscle) and trees in MEGA 6.0 (http://www.megasoftware.net) using the maximum-likelihood algorithm with 2,000 bootstrap replicates. Genetic distances were calculated in MEGA 6.0 with the General Time Reversible Gamma distributed model. Scale bars represent substitutions per site. Black arrows indicate the Peruvian isolate.
